# Hot Corrosion Behavior of Sputtered Nanocrystalline Coating with Yttrium Addition at 900 °C

**DOI:** 10.3390/ma7042882

**Published:** 2014-04-09

**Authors:** Wei Jiang, Ping Yu, Wen Wang, Shenglong Zhu, Fuhui Wang

**Affiliations:** 1 School of Materials and Metallurgy, Northeastern University, Shenyang 110819, Liaoning, China; E-Mail: jwyj5470@126.com; 2 State Key Laboratory for Corrosion and Protection, Institute of Metal Research, Chinese Academy of Sciences, Shenyang 110016, Liaoning, China; E-Mails: slzhu@imr.ac.cn (S.Z.); fhwang@imr.ac.cn (F.W.); 3 College of Information Engineering, Shenyang University of Chemical Technology, Shenyang 110142, Liaoning, China; 4 College of chemical Engineering, Shenyang University of Chemical Technology, Shenyang 110142, Liaoning, China; E-mail: yupingteacher@sina.com

**Keywords:** nanocrystalline coating, sputtering, yttrium, hot corrosion

## Abstract

The high temperature corrosion behavior of sputtered nanocrystalline K38 coating with and without yttrium addition under mixed molten salt film in air was investigated. Accelerated corrosion occurred on the coating without yttrium (Y) addition locally after 60 h exposure at 900°C, which resulted in negative weight gain in kinetics. A uniform and protective alumina scale formed on surface of the coating containing yttrium in comparison. Y enriched particle as corrosion product was observed on the top of alumina scale. The results indicated the beneficial influence of Y on the chemical stability of the protective scale in the presence of chloride. The mechanism was discussed.

## 1. Introduction

Hot corrosion induced by salts deposition is a common phenomenon for the blades and vanes in modern gas turbines, especially in seawater environment [1–3]. Molten salts deposits, such as sulfates (S), can remarkably accelerate the degradation rate of metallic materials. Moreover, the presence of chlorides in the salts can increase the corrosion rate by a factor of about 100 compared to that in sulfate salts. Therefore, the development of protective coatings is of importance to the usage of metallic materials in hot corrosion environment.

Since 1990s, extensive works showed that nanocrystalline coating, deposited by means of sputtering, exhibits excellent resistance to both high temperature oxidation and hot corrosion [4–7]. It is well known that the active element is beneficial to the stability of protective oxide scale, especially in the S containing environment. Similar positive effect was observed on nanocrystalline coatings [7]. However, few works were carried out to investigate the synthetical effect of the active element and nanocrystallization. Our previous works revealed the addition of 0.5 wt.% yttrium (Y) in sputtered K38 coating was negative to oxidation resistance in air [8], but beneficial to corrosion resistance in molten sulfate salts [9]. The chemical reaction between Y and S was considered as the main reason for the beneficial effect of Y. However, no apparent evidence was observed on coating samples to support that speculation in the previous work. In order to reveal the role of Y, corrosion behavior of sputtered nanocrystalline K38 coating containing 0.5 wt.% Y under molten salts film was investigated in this work.

## 2. Results

**Figure 1.**
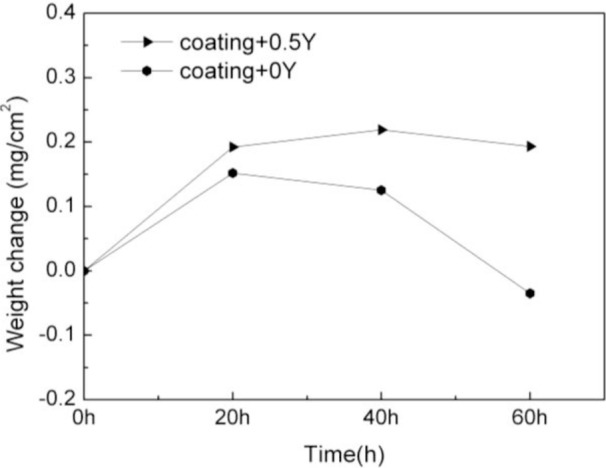
Corrosion kinetics of the sputtered coatings with the deposits of salt containing 95 wt.% Na_2_SO_4_ and 5 wt.% NaCl at 900°C.

The corrosion kinetics of the sputtered coatings with Na_2_SO_4_-NaCl film at 900°C was shown in Figure 1. It can be seen that the weight of coating K38-0Y increased during the initial 20 h Extending corrosion time caused apparent weight loss and negative weight gain was obtained after 60 h exposure. As a comparison, the weight of coating K38-0.5Y gradually increased in 40 h, slight weight loss was observed after 60 h corrosion. Corrosion of the coatings in molten sulfate for 100 h at 900°C did not show any noticeable weight loss [9], which implied that chloride promoted scale spallation.

**Figure 2.**
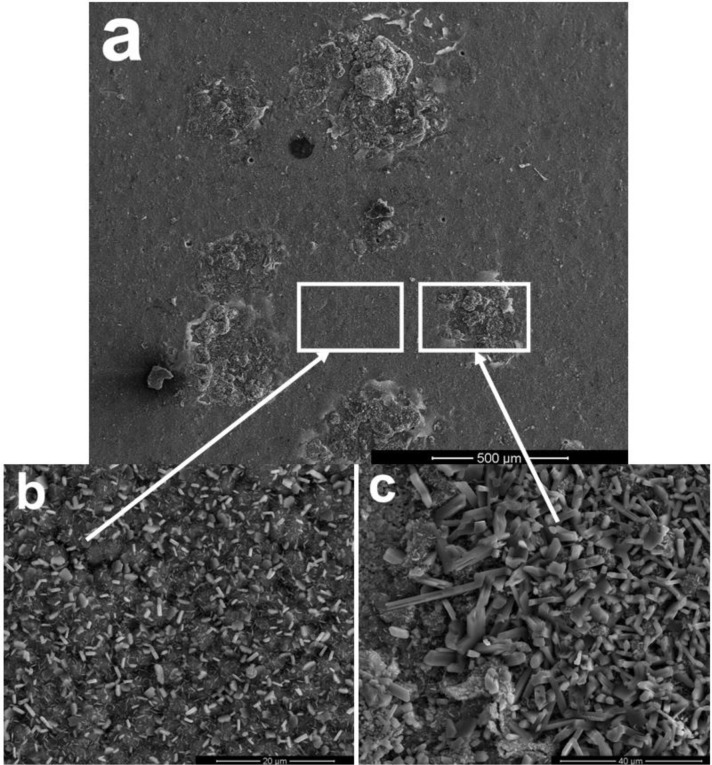

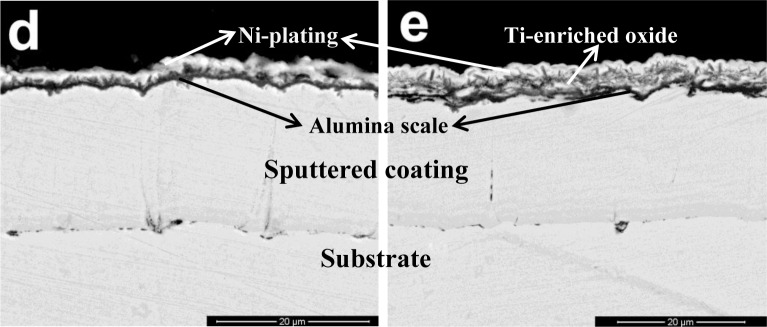
Scale morphology of coating K38-0Y after 60 h exposure at 900°C: (**a**) surface morphology, (**b**) magnified view of surface morphology of alumina scale, (**c**) magnified view of spalled area on the surface, (**d**,**e**) are the cross-sectional morphologies corresponding to (**b**,**c**).

Figure 2 shows the scale morphology of K38-0Y coating after 60 h exposure with the presence of Na_2_SO_4_-NaCl film at 900°C in air. Local spallation of scale apparently occurred, as shown in Figure 2a. Combining XRD and EDS results, it can be seen that alumina scale formed on the coating surface with fine lathy Ti-enriched product on the top (Figure 2b,d). More and coarser Ti-enriched corrosion product was observed in the spallation area, and a continuous Al rich oxide layer formed at the scale/coating interface (Figure 2c,e). No sulfide was found in the coating beneath the Al-enriched oxide layer within the detection limits, which implied that the nanocrystalline coating had excellent self-healing ability.

**Figure 3.**
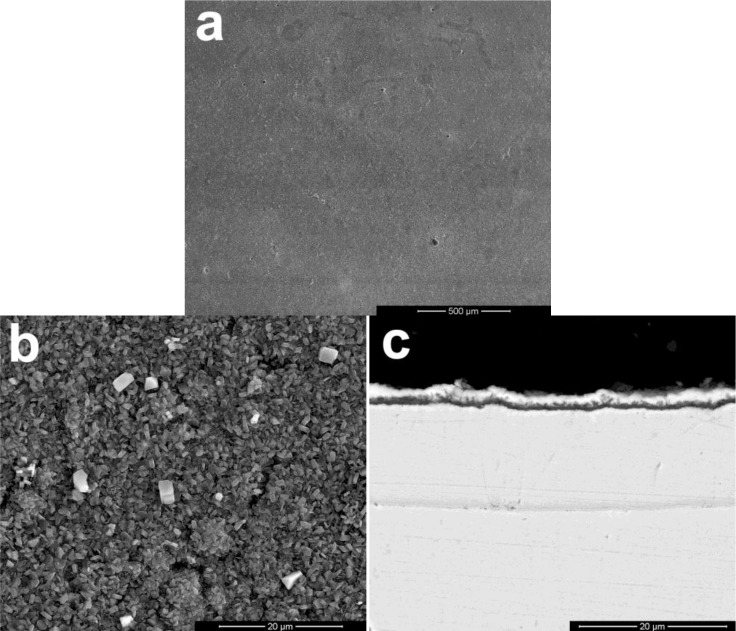
Scale morphology of coating K38-0.5Y after 60 h exposure at 900°C: (**a**) surface morphology, (**b**) magnified view of (**a**), (**c**) the cross-sectional morphology corresponding to (**b**).

**Figure 4.**
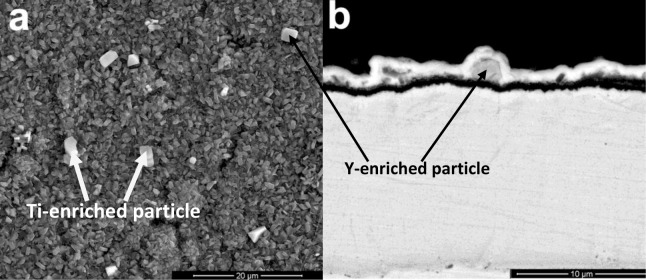
The (**a**) surface and (**b**) cross-sectional morphology of Y-enriched particle formed on the K38-0.5Y coating surface after 60 h at 900°C.

**Figure 5.**
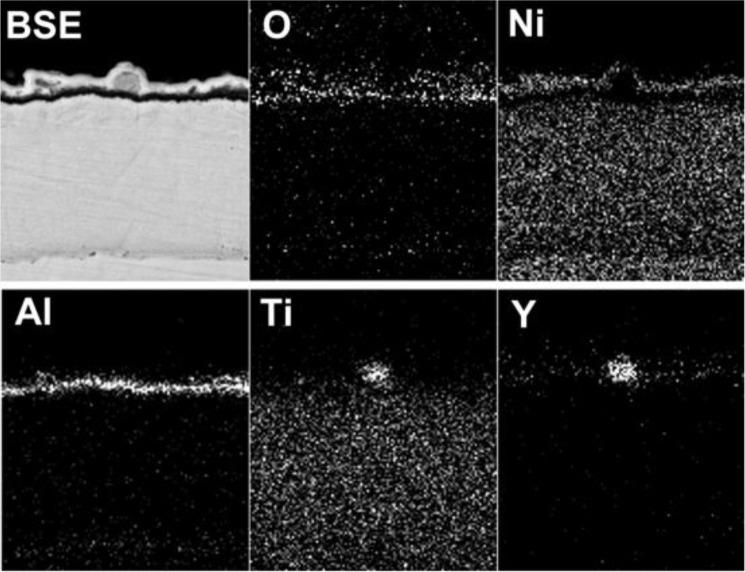
XRD Elemental mapping of the Y-enriched particle formed on the K38-0.5Y coating surface at 900°C.

The scale morphology of coating K38Y-0.5Y was comparatively uniform, as shown in Figure 3. XRD and EDS analysis revealed that thin protective alumina scale formed with crystal Ti-enriched particle scattered on the top surface. Neither sulfidation of the coating nor scale spallation was observed after 60 h exposure. The scale morphology was similar to that formed in deep molten sulfate [9]. However, particles enriched in Y on the top of alumina scale was found in this work, as shown in Figure 4, although there is difficulty to distinguish them from those Ti-rich particles by morphology. The elemental mapping analysis indicated the particles were enriched in both Y and Ti, which can be seen in Figure 5.

## 2. Experimental Procedures

The nominal chemical composition of K38 alloy is 0.1~0.2C, 15.7~16.3Cr, 8.0~9.0Co, 2.4~2.8W, 1.5~2.0Mo, 3.2~3.7Al, 3.0~3.5Ti, 0.6~1.5Nb, 1.5~2.0Ta, 0.05~0.15Zr, Ni balance (in weight percent). The sputtering targets with 0 and 0.5 wt.% (nominal content) yttrium additions were melted in a vacuum-induction furnace and then machined into dimension of 380 mm× 126 mm× 10 mm. The coatings were deposited by magnetron sputtering and the detailed sputtering parameters were described in literature [10]. Cast K38 alloy is used as substrate in order to eliminate the influence of interdiffusion between coating and substrate. Coated samples are denoted as K38-0Y and K38-0.5Y corresponding to the yttrium additions of 0 and 0.5 wt.% respectively. The thickness of coatings is around 20 μm.

The coupons with the dimension of 25 mm × 15 mm × 3 mm were coated with salt by spaying aqueous solution containing Na_2_SO_4_ and NaCl with the sulfate/chloride ratio on weight basis equal to 19:1, then evaporating the water to achieve 0.8–1 mg/cm^2^ salt film. Corrosion test was carried out at 900°C in air, and coupons were taken out at certain intervals. After cooling to room temperature, the salts were removed by boiling water followed by drying and weighting. Then, the coupons were re-heated in furnace after re-deposition of fresh salt.

The coupons after corrosion test were characterized using scanning electron microscopy (SEM) with energy-dispersive X-ray analysis (EDS) (FEI, Hillsboro, OR, USA) and X-ray diffraction analysis (XRD) (PANalytical, Almelo, the Netherlands).

## 4. Discussion

Our previous work [9] showed that the cast K38 alloy suffered basic fluxing in molten sulfate induced by sulfidation. Meanwhile, nanostructure obviously improved hot corrosion resistance by promoting the rapid formation of protective alumina scale. The addition of Y was considered to further prevent the sulfidation of nanostructured coating since Y acts as the “gettering” of S. However, no evident segregation of Y was observed in the scale or at the scale/coating and scale/salt interfaces in that work. It is well known that the presence of chloride increases the propensity of protective scale to crack and spall [11], although the role of chlorides in hot corrosion is not well understood. Metallic chlorides are supposed to form during corrosion, which have relatively high vapor pressures compared to metallic sulfates. The gaseous metallic chlorides move outward through the salt film and are converted to non-protective metallic oxides at salt/gas interface, the chlorine is thereby recycled to react with alloying elements in the metal. Continuation of this process results in rapid material degradation [12,13]. In the present work, scale spallation apparently occurred on the K38-0Y coating in the presence of both chloride and sulfate. Non-protective Ti enriched corrosion product formed in the spalled area, nevertheless the formation of protective scale at the scale/coating interface beneath the non-protective product prevented further sulfidation and chloridation of alloying elements of the coating. This result indicated the excellent self-healing ability of the sputtered nanocrystalline coating. Comparatively, no evident cracking/spallation of scale was observed on the K38-0.5Y coating. Thin, protective alumina scale formed on the coating surface, the scattered Ti-enriched particles should form at the initial stage of corrosion before the formation of continuous protective scale. The formation of Y-enriched particles on the top surface of alumina scale might evidence the participation of Y in the corrosion reaction. The melting and boiling points of YCl_3_ are relatively high among the chlorides listed in Table 1 [14]. Usually, high melting and boiling points predicate high bonding energy and low vapor pressure. Therefore, yttrium may affect the hot corrosion of the coating in two ways. The chloridation of Y or Y-oxide due to the chemical affinity between Y and Cl could decreased the activity of Cl at the scale/salt interface, thus promoted the chemical stability of protective alumina scale. Secondly, the higher boiling point of Y-chloride might result in relatively slow transport rate of Y-chloride from scale/salt interface toward salt/gas interface, which thus would retard the counter-diffusion of Cl to the scale/salt interface. Both the two mechanism could improve the chemical stability of protective alumina scale on the coating surface.

## 5. Conclusions

The hot corrosion of sputtered nanocrystalline K38 coatings with and without Y addition was investigated with Na_2_SO_4_ deposit containing 5 wt.% NaCl at 900°C. The nanostructured coating exhibited excellent self-healing ability although scale spallation occurred resulted from the presence of chloride. No negative effect of chloride was apparently observed on the coating coupon with Y addition after 60 h exposure. Y-enriched particles formed on the alumina scale, which indicated the beneficial effect of Y on the chemical stability of protective scale in the presence of chloride.

## Figures and Tables

**Table 1.** The melting and boiling points of selected chlorides.

Physical Properties	Chlorides					
	YCl_3_	AlCl_3_	CrCl_2_	CrCl_3_	TiCl_3_	TiCl_4_
Melting point (°C)	721	192	824	1152	425*	−24
Boiling point (°C)	1507	–	1302	1300*	960	136

* decomposes.
